# FGM and Restorative Justice—A Challenge for Developing Countries and for Refugee Women

**DOI:** 10.3390/ijerph18178913

**Published:** 2021-08-24

**Authors:** Thomas Wenzel, Jan Ilhan Kizilhan, Reem Alksiri, Daniela Dörfler, Eva Jana Messerschmidt, Anthony Fu Chen

**Affiliations:** 1 World Psychiatric Association Scientific Section on Psychological Aspects of Persecution and Torture, 1226 Geneva, Switzerland; remarom73@gmail.com (R.A.); anthonyfchen@gmail.com (A.F.C.); 2 CEHRI, The Centre for the Enforcement of Human Rights International, Schwarzspanierstraße 15/1/17, 1090 Vienna, Austria; 3 Duale Hochschule Baden-Württemberg, 78054 Villingen-Schwenningen, Germany; kizilhan@dhbw-vs.de; 4 University of Dohuk, AJ Duhok 1006, Iraq; 5 Department Gynaecology, Medical University of Vienna, 1090 Vienna, Austria; daniela.doerfler@meduniwien.ac.at; 6 Independent Researcher, 1010 Vienna, Austria; office@messerschmidt.lawyer

**Keywords:** female genital mutilation, refugees, migrants, human rights, gender, transitional justice, psychological trauma, gynecology, primary mental health

## Abstract

Female Genital Mutilation (FGM) has been identified as one of the most serious human rights violations women are exposed to in many countries, in spite of national and international efforts. The actual implementation of preventive strategies and support of victims faces a number of challenges that can only be addressed by an interdisciplinary approach integrating public health and legal considerations. FGM in the context of women as refugees who left their country to escape FGM has rarely been covered in this context. This article summarizes the most important international standards and initiatives against FGM, highlights the medical, legal, and psychological factors identified so far, and explores the interdisciplinary considerations in changing a country and society to permit safe return of those escaping FGM to third countries and support public health in the country.

Increasing awareness of the harms of female genital mutilation (FGM) has been the focus of one of the most prominent, globally-concerted efforts to improve the human rights situation of women, with the World Health Organization (WHO) at the forefront. In the following article we intend to explore and summarize the interdisciplinary challenges and global positions with regards to the protection and support of victims of FGM, with special consideration of refugees or prospective returnees from exile to a country with recent or ongoing FGM. The discussion cannot be limited to medical or legal considerations alone, but must consider other aspects, such as legislation and social psychology, and the experiences with difficult transitions in countries with different forms of pervasive violence. We first want to examine and summarize the WHO’s stance on FGM as reflected in fact-sheets and documents, as they can be seen as the reference standard for the international understanding of the problem, and will subsequently examine other international standards of note, such as the UNHCR recommendations of protection of victims and women at risk.

The WHO officially defines four types of FGM: Type 1: (clitoridectomy) a “partial or total removal” of the clitoris or of the prepuce, Type 2: (excision) as “partial or total removal of the clitoris and the labia minora”, with a subtype characterized by the additional excision of the labia majora, Type 3: (infibulation) as “narrowing of the vaginal opening through the creation of a covering seal”, with subtypes referring to the additional performance or absence of clitoridectomy. Type 4 summarises “all other harmful procedures to the female genitalia for non-medical purposes, e.g., pricking, piercing, incising, scraping and cauterizing the genital area.” (https://www.who.int/news-room/fact-sheets/detail/female-genital-mutilation, accessed on 12 April 2021).

The WHO summarises a number of severe immediate (short term) consequences including but not limited to “pain, haemorrhage, genital tissue swelling, fever, infections (e.g., tetanus), urinary problems, wound healing problems, injury to surrounding genital tissue, shock”, and even death, any of which should prohibit the practice.

Long-term consequences beyond the obvious severe psychological and physical suffering can include (again using the WHO as a reference): painful urination, urinary tract infections, vaginal discharge, itching, bacterial vaginosis and other vaginal infections, painful menstruations, scar tissue and keloid, pain during intercourse, decreased satisfaction. Further WHO mentions include childbirth complications such as a “difficult delivery”, and further risks to the mother including “excessive bleeding, caesarean section”, and child including the need for resuscitation or even newborn deaths. Further considerations include the risk and stress associated with later, often repeat, surgical interventions such as deinfibulation, which cause pain, incur cost, and can lead to risks to life and health of the survivor. Psychological problems are the final but significant complication and include anxiety, depression, post-traumatic stress disorder, and more general psychological sequelae such as low self-esteem, as listed again by (WHO, (https://www.who.int/news-room/fact-sheets/detail/female-genital-mutilation, accessed on 12 April 2021)) and also by other authors [1,2,3].

The WHO authors therefore concluded: “FGM has no health benefits, and it harms girls and women in many ways” (Female Genital Mutilation/Cutting: A Global Concern, UNICEF, New York, 2016, https://data.unicef.org/resources/female-genital-mutilationcutting-global-concern/, accessed on 23 August 2021), and the World Health Assembly consequently passed the 2018 resolution WHA61.16 “regarding the elimination of FGM, emphasizing the need for concerted action in all sectors including health, education, finance, justice and women’s affairs” (https://reliefweb.int/report/world/female-genital-mutilation-enarruzh, (accessed on 12 April 2021)).

A special day, February 6th every year, has even been declared “International Zero Tolerance for Female Genital Mutilation (FGM)” Day.

Not only are there global intervention and advocacy programs to prevent FGM, but there is a further argument that the existence of FGM is justification for asylum, as it is an important reason for women to seek protection or a safer life in another country to escape severe physical and psychological harm [4]. Even by 2013, the UNHCR had published a comprehensive report on FGM and asylum in the EU (FEMALE GENITAL MUTILATION & ASYLUM IN THE EUROPEAN UNION, UNHCR, Geneva 2013, https://www.unhcr.org/531880249.pdf, accessed 23 August 2021). According to this report, “about 20,000 women and girls seek asylum from FGM-practicing countries of origin in the EU every year. This number has remained relatively constant between 2008 (18,110) and 2011 (19,565), despite the total number of female applicants having increased from 65,125 in 2008 to 93,350 in 2011 due mostly to the general reduction in asylum claims from Somalia; Somali women and girls represented about 20% of all female applicants in 2011”.

Unfortunately, to the best of our knowledge, no similar updated report has been released, which underlines the need for further global research and monitoring research in this area. FGM is in this context also accepted as a criterion for special protection needs, and victims are classified as a “vulnerable group” in the EU reception guidelines, which should receive proper identification and support (European Union: Council of the European Union, Directive 2011/95/EU of the European Parliament and of the Council of 13 December 2011 on standards for the qualification of third-country nationals or stateless persons as beneficiaries of international protection, for a uniform status for refugees or for persons eligible for subsidiary protection, and for the content of the protection granted (recast), hereinafter EU Qualification Directive (Recast), 20 December 2011, OJ L 337; December 2011, Art. 4(2) and 4(3), available at: http://www.unhcr.org/refworld/docid/4f197df02.html40, (accessed on 12 April 2021) EU Qualification Directive (Recast), Art. 9(2)(f), accessed April 12, 2021). UNHCR observes that asylum acceptance rates differ significantly and draws attention to the fact that some countries gave protection to only small numbers of women and girls seeking asylum from FGM-practicing countries of origin (mentioning Italy, n = 75 for the period between 208 and 2011), and numbers differed significantly. For example, Austria granted protection to 145 women and girls, which was more than Belgium and Italy. On the other hand, only Belgium gathers systematic data on FGM mentioned as a reason to apply in asylum claims. Apparently, EU states deal in different ways with this situation. Several recent reviews have addressed the role of medical evaluation of FGM in asylum cases [4,5]. A more recent study by van Baelen et al. used data from the EU census not limited to refugees to demonstrate that more than half a million first-generation women and girls in the EU, Norway and Switzerland had suffered FGM before immigration [6].

Reception in host countries is characterized by a number of challenges, summarized in the above-mentioned UNHCR report, and include language, limited cultural awareness and expertise. UNHCR recommended well-coordinated inter-agency trainings to provide effective and adequate support and become aware of and react to early warning signs, especially in children (as above, citing “Female genital mutilation, asylum seekers and refugees: the need for an integrated European Union agenda”, Richard A. Powell, Els Leye, Amanda Jayakody, Faith N. Mwangi-Powell, Linda Morison, Health Policy. 2004 Nov;70(2):151-62, doi:10.1016) and in maternity care [7].

The above report has no data on the fate of those who were refused asylum or returned to the FGM-practicing countries of origin by force or their own free will. Individuals seeking asylum on FGM-related grounds can qualify for refugee status under the 1951 Refugee Convention or other forms of international protection, if the authorities in the countries of origin are unable or unwilling to protect them effectively from the practice. (see for example the UNHCR Guidance Note on Refugee Claims Relating to Female Genital Mutilation, May 2009, https://www.refworld.org/pdfid/4a0c28492.pdf, accessed 12 April 2021. It is well-established that victims and potential victims of FGM can be considered as members of a particular social group.) UNHCR holds that “all forms of FGM are considered harmful and violate a range of human rights, as affirmed by international and national jurisprudence and legal doctrine”, and that FGM amounts to persecution. (UNHCR, Handbook on Procedures and Criteria for Determining Refugee Status, February 2019, p. 153., online at https://www.unhcr.org/4d93528a9.pdf, accessed 23 August 2021) In principle, a person that has been granted refugee status or other forms of protection based on FMG-related grounds can only be returned if the reason to seek asylum has been resolved, for example indicating that “substantial changes including legal prohibition and effective prevention is provided”, which is clearly not the case in many countries. Such substantial changes cannot be achieved overnight and might require, as already mentioned, an interdisciplinary approach integrating medical, psychological, and social policy together with legal interventions.

In the following, we are not arguing against the need to abolish FGM quickly and effectively, given its severely adverse medical and psychological impacts, but we do want to summarize and discuss the far-reaching changes that are required in society to abolish FGM and create a safe and supportive process for all social groups. This must also be a precondition before any women can be returned to the countries they left for FGM-related reasons.

Most refugees only leave their countries out of necessity, such as war, or the threat of human rights violations such as FGM. Many desire a return to their homes, friends, and family, but they require a clarification of the status of safety including persistence of FGM: Does the danger persist? Is there impunity for perpetrators? Can I expect to be respected and supported back home? Will I get financial support and adequate medical and psychological treatment?

Changing a society’s views and its legal system regarding such a widespread issue is obviously not an easy task. A number of books have been published documenting the—often very successful—efforts to not only ban FGM, but also change the psychological and legal frameworks in more traditional societies where FGM has a long-standing history and might be embedded in cultural belief systems [8,9,10], thus creating a barrier to change. Comprehensive solutions are usually recommended [11,12], as formal legislation and sanctioning have the limitation of insufficient community support in poorer, “developing” traditional culture countries (as also noted in the WHO publications listed above), and cannot be expected to change the situation for present or prospective victims in a reasonable time.

What further challenges will be encountered and should be considered when eliminating FGM from a society? How can it make the country of origin safer and acceptable for the possible return of past FGM victims and those otherwise exposed to the risk of FGM on return?

The social consensus in such societies to stigmatize women who have not complied with FGM [13] as well as against family members who do not condone or help to implement the practice, clearly needs to be addressed, as noted by Brown [14] and other authors. The WHO provides educational materials, including an excellent PowerPoint series, that can be used to advocate against FGM, but a number of recent books have demonstrated how complex successful intervention projects can be (see for example Burrage [15]).

There is also a substantial body of publications on the legal aspects of FGM as part of public health, including on the legislation in host countries such as in the EU [14], but also in Africa [16]. Two recent but well-established closely-related concepts can help to offer possible solutions to a situation that arises when a common practice in a changing society that was once accepted is now declared a human rights violation, as this shift in attitude suddenly creates a challenge of how to deal with large numbers of past and present perpetrators and victims and their interaction in society. A community-based approach is therefore required to address the problem in a situation where lawyers, medical doctors and healthcare providers, families, and the legal institution could be seen as possible perpetrators or “crime” affiliates, but have to live in the same society together with the victims of FGM, even when the practice has been officially abolished.

“**Restorative justice**” is a new concept usually employed after severe Human Rights violations in post-conflict countries to address impunity, while “**transitional justice**” (TJ) describes the process of change necessary to rebuild civil society after severe human rights violations or a breakdown or “rule of law” [17]. Both concepts are relevant for the present situation in regard to FGM, especially in African countries. In one of the most elaborate systematic discussions so far, Pathak (2017) proposed that transitional justice “constitutes a five-pillar approach: truth, justice, healing, prosecution and reparation” (https://www.transcend.org/tms/2020/09/nuremberg-tribunal-a-precedent-for-victors-justice/, accessed on 12 April 2021).

Restorative justice is usually also seen as a positive and necessary step in the psychological recovery of groups and individuals, and it is known as an interdisciplinary challenge (see also Reiter, [18]). It includes the consideration of legal, economical, sociological, medical, psychological, and cultural factors and frequently makes use of culture based arbitration and justice models provided by traditional local cultures [19], such as Gacaca courts in Rwanda [20].

The few studies published demonstrate that though the impact of TJ is hard to assess, results in countries such as South Africa or Rwanda are not unequivocal [20]. It has also recently been explored in regard to Nepal [21] and flaws have been identified that make an efficient process in some situations impossible; the summary report by the independent International Commission of Jurists (ICJ) organization concluding that “COIs (Note: Commissions of Inquiry) have promoted impunity by diverting investigation of human rights violations and crime from the criminal justice process into a parallel ad hoc mechanism vulnerable to political interference and manipulation” [21].

Still, few alternatives exist, and the models need to be improved and explored by further research to identify effective models based on these concepts.

We propose that the following challenges as outlined in Table 1 can be expected when changing to an “FGM-free” society:

The still ongoing discussion reflecting a conflict between international or secular legal standards, culture and traditional Scharia law in Egypt on the FGM issue can serve as an example on the above issues [22,23], though to our knowledge no data are available so far on the public health and economic impact of the different solutions. The above reasons obviously do not apply to the continued use of FGM in host countries with long-standing existing legislation against FGM or similar acts where the influx of migrants from FGM practicing countries might create different critical situations to be explored later on in this article. Traditional healers, and other persons that perform circumcision including midwives without adequate formal surgical training should also be considered in this context. The study by Dattijo, for example, showed that 31.3% of a sample of expecting mothers in a teaching hospital in Nigeria had suffered FGM, and in most cases it was reportedly performed by traditional healers [24]. This underlines again that it is important to find solutions that fit to the environment of traditional cultures. Omar et al. [25], who explored this problem in a paper on the risks of HIV infection by FGM in Somali nomads, asked for more research, in this case also on pastoralist-specific policies in regard to addressing FGM and its risks. Myths and false health belief systems must obviously be addressed in this context [26,27].

A further issue with returning FGM victims to their countries of origin is the lack of adequate medical care due to limited “in-country training” of local health care professionals, let alone the fact that there are substantial financial barriers victims face to access such treatment even when it is available. The inadequate actual access in everyday practice to necessary treatment must also be seen as a reason against forced return of victims of FGM. Forced return to the country of origin may in this case also violate the principle of non-refoulement in international law, according to which persons must not be returned while there is a serious risk that he or she will be subjected to relevant human rights violations.

All of these listed considerations illustrate that a comprehensive solution must be first implemented and demonstrated to actually work in daily, “living” practice before return can be considered. Imprisonment of for example doctors who have practiced or are still practicing FGM is frequently a problematic solution, only addressing two of the “pillars” mentioned above. It can create a substantial list of secondary problems such as the risk to healthcare provision in the already insufficient healthcare networks if doctors and other healthcare workers are taken to prison and therefore out of their communities. If, for example, the main family member responsible for taking a child to FGM is imprisoned, the impact on the family income and functioning can be potentially severe and damaging.

Economic sanctions (punitive damages) serve to punish and are frequently employed as alternative punitive and preventive interventions together with or without imprisonment, and might also serve to demonstrate that an act committed was “wrong” and is not accepted by society. However, if they are to be paid by healthcare workers or family members, it would potentially again cripple the functioning of the often uninsured healthcare experts, or the families themselves. Traditional healers and other persons performing FGM must, as noted before, also be reached by any intervention.

Recompense of necessary treatment costs must be seen as a main goal, as most countries with widespread FGM practices do not have comprehensive free healthcare plans in public health.

These may be less of a problem in cases where healthcare professionals receive a substantial income through FGM or by other means, and it could be proposed that those profiting from illegal FGM should contribute to resolution of the damage caused by their actions, at least if FGM was illegal at the time it was performed.

As with any prohibition, shifting the classification of FGM to the category of being illegal and medically dangerous must take into account the impact on alternative “providers” such as local healers, if no comprehensive solution is provided.

The already overwhelming financial burdens and low capacity of both healthcare experts and family members obviously make both imprisonment and the taking of financial responsibility as noted before very difficult, especially when FGM was previously permitted by law. These might also, as already outlined, damage the victims and create secondary victims in the form of family members punished, which may outweigh the preventive impact. Further, it must be assumed that neither can actually contribute to finance the special treatment necessary for FGM victims in a developing country, even when available.

The need for justice, addressing impunity, and prevention must therefore in our opinion be carefully balanced with the actual in-country situation and the best interests of the victims and society without creating a situation of impunity.

Being forced to return to an unresolved situation would obviously endanger the physical and psychological health of past and potential victims living as refugees.

Possible solutions to the dilemmas of transitional justice after FGM (and similar severe human rights violations).

Similar problems arise with other crimes in traditional societies, especially in Africa, and traditional justice models stressing alternative solutions to imprisonment or to high punitive damages have been proposed. This has also been recognized in other regions such as the EU where the Committee of Ministers of the Council of Europe issued a recommendation to member states which recognized “the potential benefits of using restorative justice with respect to criminal justice systems” and encouraged member states to “develop and use restorative justice” (“Recommendation CM/Rec(2018)8 of the Committee of Ministers to member States concerning restorative justice in criminal matters”. 3 October 2018. Retrieved 7 March 2019)

The Acholi Mato Oput ceremony in Uganda can be seen as an example in this context [28] and has been used for example in the reintegration of child soldiers [28].

Community-based models and those using traditional resolution models that have a high acceptance rate and practicability should therefore be considered, though this might not always work out easily, for example looking again at the adaptation of Gagacha courts in Rwanda [20], which attempted to address the impunity of perpetrators of the genocide [20]. The use and adaptation of such existing models, supported by well accepted social media channels to serve the process of transition could be important components of an integrated strategy in traditional societies.

Regarding financial compensation or support for treatment of victims, local governments and the international community are the major options, as neither family members nor local healthcare experts have the financial capacity to recompense for treatment, even if they have been found guilty. This is similar to the approach in the EU framework on victims of crime support (Directive 2012/29/EU of the European Parliament and of the Council of 25 October 2012 establishing minimum standards on the rights, support and protection of victims of crime, and replacing Council Framework Decision 2001/220/JHA) that underlines the states responsibility to step in with support in different forms when access to treatment would otherwise be dependent on the uncertain outcome of prosecution and a court case.

Finally, the local governments and international community will need to build the capacity and competence for state-of-the art treatment in countries of origin. While physical health long-term impact as outlined by the WHO must be treated, psychological impact should not be neglected. For example, one of the authors of this article had documented the substantial psychological impact of FGM in 79 circumcised Kurdish girls between 8 and 14 years of age in 2011. Thirty uncircumcised girls from the above area and 31 uncircumcised girls from other areas of Iraq served as comparison subjects. Sequels included higher rates of PTSD (44.3%), depression disorder (33.6%), anxiety disorder (45.6%), and somatic disturbance (36.7%) in the circumcised group [29] and no adequate treatment resources offered by trained mental health care experts were available at the time.

Forcing a victim to return is unacceptable by international standards if no adequate treatment can be provided in countries of origin but would be available in host countries. In this context, the principle of non-refoulement, as referred to above, must be upheld.

Changing the perception of the act of FGM and of victims in a society is also an important precondition to any return, beyond simple prohibition and effective provision of adequate medical services, as a hostile or ambivalent reaction or stigma might lead to secondary victimization even if FGM is now prohibited, as demonstrated in a recent study the authors performed for UNICEF in the case of families forcefully returned to Kosovo. Untreated psychological trauma was common, could not be treated by the low resources of the “repatriate” country, and returned children were stigmatised and isolated in the local communities (Available from https://www.unicef.org/.../SILENT_HARM_Eng_Web.pdf, accessed on 27 May 2019).

## Conclusions

Though there has been progress made in banning FGM in many regions, legal prohibition cannot be seen as sufficient for a safe environment on the ground for the return of refugees who have experienced or were threatened by FGM. Strategies based on culture-sensitive community interventions supported by legal models based on restorative and transitional justice should be considered as the main or as supportive measures when the usual punitive legal models cannot be implemented without risk of further damage to earlier victims or indirect victims such as family members. Governments and international NGOs must be instrumental in providing medical treatment and rehabilitation independent from civil law based concepts directly asking for compensation and liability requested by the perpetrators- family, and health care professionals.

## Figures and Tables

**Table 1:** Challenges in countries of origin.

	Medical Doctors and Traditional Healers	Family Members
Motivation barriers	Pressure by family members and society, lack of awareness, financial interests, interest to prevent unprofessional interventions	Pressure by family members and society, lack of awareness
Indemnity for pain experienced	“post-hoc” legislation (no liability), could endanger continuous work for other patients as doctors already have low income	“post-hoc” legislation cannot be applied if FGM was legal
Indemnity for present psychological/physical impairment	“post-hoc” legislation (no liability), could endanger continuous work for other patients, low income	“post-hoc” legislation cannot be applied if FGM was legal
Sanctions	Threatening imprisonment will contribute to brain drain and endanger already fragile health systems, might create tensions and split in societies	Imprisonment of economical or care providers in the family will endanger already economical and in general fragile family systems

## Data Availability

Not applicable.
